# Incidence of nephrotoxicity associated with intravenous colistimethate sodium administration for the treatment of multidrug-resistant gram-negative bacterial infections

**DOI:** 10.1038/s41598-022-19626-2

**Published:** 2022-09-10

**Authors:** Svetlana Sadyrbaeva-Dolgova, Ricardo García-Fumero, Manuela Exposito-Ruiz, Juan Pasquau-Liaño, Alberto Jiménez-Morales, Carmen Hidalgo-Tenorio

**Affiliations:** 1 Pharmacy Department, Hospital Universitario Virgen de las Nieves, Av. Fuerzas Armadas, 2, 18014 Granada, Spain; 2 Infectious Disease Department, Hospital Universitario Virgen de las Nieves, Granada, Spain; 3 Instituto de Investigación Biosanitaria ibs.GRANADA, Granada, Spain; 4 Pharmacy Department, Hospital Universitario de Gran Canaria Dr. Negrín, Las Palmas, Spain; 5 Unit of Biostatistics, Department of Statistic and Operational Research, School of Medicine, University of Granada, Granada, Spain

**Keywords:** Bacterial infection, Antibiotics, Antimicrobial resistance

## Abstract

Colistimethate sodium (CMS) is the inactive prodrug of colistin, CMS has a narrow antibacterial spectrum with concentration-dependent bactericidal activity against multidrug-resistant gram-negative bacteria, including *Pseudomonas aeruginosa* and *Acinetobacter baumannii.* This study aimed to analyze potential correlations between clinical features and the development of CMS-induced nephrotoxicity. This retrospective cohort study was conducted in a tertiary-care university hospital between 1 January 2015 and 31 December 2019. A total of 163 patients received CMS therapy. 75 patients (46%) developed nephrotoxicity attributable to colistin treatment, although only 14 patients (8.6%) discontinued treatment for this reason. 95.7% of CMS were prescribed as target therapy. *Acinetobacter baumannii* spp. was the most commonly identified pathogen (72.4%) followed by *P. aeruginosa* (19.6%). Several risk factors associated with nephrotoxicity were identified, among these were age (HR 1.033, 95%CI 1.016–1.052, *p* < 0.001), Charlson Index (HR 1.158, 95%CI 1.0462–1.283; *p* = 0.005) and baseline creatinine level (HR 1.273, 95%CI 1.071–1.514, *p* = 0.006). In terms of in-hospital mortality, risk factors were age (HR 2.43, 95%CI 1.021–1.065, *p* < 0.001); Charlson Index (HR 1.274, 95%CI 1.116–1.454, *p* = 0.043), higher baseline creatinine levels (HR 1.391, 95%CI 1.084–1.785, *p* = 0.010) and nephrotoxicity due to CMS treatment (HR 5.383, 95%CI 3.126–9.276, *p* < 0.001). In-hospital mortality rate were higher in patients with nephrotoxicity (log rank test *p* < 0.001). In conclusion, the nephrotoxicity was reported in almost half of the patients. Its complex management, continuous renal dose adjustment and monitoring creatinine levels at least every 48 h leads to a high percentage of inappropriate use and treatment failure.

## Introduction

Severe nosocomial infections due to multidrug-resistant gram-negative bacteria (MDR-GNB) are deemed to be of high morbidity and mortality. The emergence of highly resistant gram-negative bacteria, in particular carbapenem-resistant *Acinetobacter baumannii* (CRAB) strains, multidrug-resistant *Pseudomonas aeruginosa* and carbapenem-resistant *Klebsiella* species, and the lack of effective antimicrobials, have led to reuse polymyxins, antibiotics available since 1950 but abandoned owing to their nephrotoxicity.

Colistin E is a polypeptide belonging to the polymyxin family, produced by *Bacillus polymyxa* subspecies *colistinus*^1^*.* Colistin’s structure includes a polycationic peptide ring composed of 10 amino acids and a hydrophobic fatty acid tail^2^. It consists of at least 30 components, the 2 major ones being colistin A and colistin B^3^.

Colistimethate sodium (CMS) is the inactive prodrug of colistin, a polymyxin that can be administered parenterally or by inhalation. CMS undergoes a rapid hydrolysis to methanesulfonated derivatives and to colistin. It is shown that colistin concentrations increase slowly after CMS administration in critically ill patients and it takes 2 days to reach steady state, suggesting the benefits of initiating treatment with a loading dose^4,5^.

CMS has a narrow antibacterial spectrum with concentration-dependent bactericidal activity against MDR-GNB, including *P. aeruginosa* and *A. baumannii*. The mechanism of action of CMS involves interaction between the polycationic portion and the anionic portion of lipopolysaccharides (LPS) that compound the outer membrane, disrupting the outer membrane of gram-negative bacteria and providing bactericidal activity and probable enhancement of the activity of other antimicrobials^6^. Numerous antibiotics have shown synergistic activity with colistin in vitro; the most studied are rifampicin and imipenem, but also ceftazidime^7,8^.

The most common adverse reaction of CMS is nephrotoxicity and neurotoxicity. Nephrotoxicity usually occurs within a median of 2.5–10 days of therapy^9–11^ and renal function usually returns to normal within 3–9 weeks after treatment discontinuation^12^. Colistin induces tubular damage by increasing the membrane permeability of epithelial cells, which increases influx of cations, anions and water, leading to leakage of contents and cell death. The proposed mechanism is related to its mechanism of action against gram-negative bacteria^9^ and has shown a dose-dependent effect^11,13^. Given that nephrotoxicity is a well-known predictor of mortality^14^, it has been a common measurement during CMS therapy. However, the incidence of renal function alterations in this context is not well-established yet as many unexplored risk factors might be involved. It is clear that an urgent strategy to properly use CMS therapy against MDR-GNB is needed, as the incidence of colistin-resistant gram-negative bacteria, especially *Acinetobacter spp.*, is increasing^15^. However, CMS therapy may still be an option for treating colistin-resistant CRAB infections^16^, increasing interest in studying the use of this valuable antimicrobial in daily practice.

Unfortunately, it is difficult to implement a patient-based approach to select the most appropriate antimicrobial therapy against these microorganisms. Interpatient variability is extensive even at a given creatinine clearance^13^, but minimizing it is crucial to enhance survival by reducing CMS-associated nephrotoxicity. This study aimed to analyze potential correlations between clinical features and the development of CMS-induced nephrotoxicity and to describe colistin therapeutic use and clinical outcomes. Results may provide a more thorough understanding of that interpatient variability to avoid CMS misuse.

## Materials and methods

This retrospective cohort study was conducted in an 800-bed tertiary university hospital in Spain between 1 January 2015 and 31 December 2019.

All patients enrolled in this study (≥ 18 y.o.) have received intravenous CMS for at least 48 h. Clinical data were gathered using the hospital electronic medical records, which contain demographic, microbiological and prescription information. Demographic and clinical characteristics of patients, including age, sex, and baseline Charlson Comorbidity Index score were included. In relation to antibiotic treatment, data were gathered related to colistin loading dose and duration, concomitant use of antibiotic, infection sites and organisms with susceptibility. Also, baseline, peak creatinine and glomerular filtration (CKD-EPI) during colistin therapy, albumin, hemoglobin and leukocytes were collected. Only one treatment per patient and parenteral administration of the treatment were considered in the analysis CMS administered by inhalation were excluded from the analysis.

Available colistin in our hospital is labelled GES® and is equivalent 1,000,000 UI (1 MU) of CMS is equal to 80 mg of CMS or 34 mg of colistin base activity (CBA). Therefore, the dose of 9 MU is equal to 300 mg and of 4.5MU is equal to 150 mg of CBA.

The main outcome variables were: all-cause in-hospital mortality rate, efficacy based on clinical and microbiological response to the treatment, and length of current hospital stays. Clinical success was defined as the resolution of symptoms and signs of infection; microbiological cure as the eradication of MDR-GNB isolates on follow-up cultures, and failure as the persistence or worsening of symptoms and “escalating” therapy with additional antimicrobial agents for this infection.

Nephrotoxicity was established following the Kidney Disease Improving Global Outcomes (KDIGO) classification: creatinine elevation of ≥ 0.3 mg/dL in 48 h or ≥ 1.5 times baseline creatinine in an interval of up to 7 days^17^. Furthermore, nephrotoxicity was attributed to colistin use, according to the criterion of the attending physician informed by the patients' medical records.

Due to the absence of any protocol for appropriate use of CMS implemented in our hospital, appropriate CMS prescription was determined as administration of a loading dose of 9 million units (MU) followed by a maintenance dose of 4.5 MU administered every 12 h or 3 MU every 8 h. In patients with moderate to severe renal impairment after a loading dose of 9 MU, maintenance doses were adjusted according to CKD-EPI clearance estimates.

Combination therapy was considered concomitant use of at least one antibiotic with activity against gram-negative microorganisms.

### Ethics approval

This study was approved by the local ethics committees (Comité de Ética de la Investigación (CEI/CEIM) de la Provincia de Granada) which also gave the waiver for requirement for informed consent. Data collection was complied with the Helsinki declaration principles and biomedical research legislation (Law 3/2018, December 5).

### Data availability

All data generated or analyzed during this study are included in this published article (and its "Supplementary information" files).

### Statistical analysis

Means, standard deviations, medians, and interquartile range (IQR) were calculated for quantitative variables, and absolute and relative frequencies for qualitative variables. Relations among qualitative variables were analyzed using Pearson’s or Fisher’s chi-square test, while quantitative variables were analyzed with non-parametric Mann–Whitney test, due to non-normal distribution (Kolmogorov–Smirnov test). Results with *p* ≤ 0.05 were considered statistically significant.

Survival analysis was performed using the Kaplan–Meier method, considering the time to occurrence of nephrotoxicity and the time to death. The probabilities of survival or nephrotoxicity development at different times, mean and median, were calculated. The log-rank test was used to compare survival between patients with and without nephrotoxicity.

Multivariate Cox regression was used to identify independent risk factors associated with nephrotoxicity and mortality. The results for both were reported as hazard ratios (HR) with 95% confidence intervals (CIs). A multivariate model was constructed using backward stepwise selection considering entry criteria of *p* ≤ 0.05 and exit criteria of *p* > 0.10. All variables that showed statistical significance in bivariate analysis or were otherwise considered relevant were included.

Data management and analysis were performed using IBM SPSS Statistics 19 software and Stata Statistical Software, version 12(College Station, TX: StataCorp LP).

## Results

### Descriptive analysis of the cohort

The study included 163 patients with CMS prescriptions over 5 years. The mean age was 66 years, and 71.2% were male.

At the time of CMS initiation, almost half of the patients were admitted to the Intensive Care Unit ICU (42.3%). The median basal glomerular filtration rate estimated according to CKD-EPI was 92.2 ml/min/1.73m^2^. The most frequent infection where CMS was prescribed was lower respiratory tract infection (41.7%) followed by urinary tract infection (22.7%) and others. 17.2% of all infections were associated with bacteremia.

Other characteristics of the patients are shown in Table 1.

**Table 1:** Baseline characteristics of patients in the study cohort and bivariate analysis of risk factors for CMS-associated nephrotoxicity.

Characteristics of patients	Total (n = 163)	Nephrotoxicity group n = 75 (46%)	Non-nephrotoxicity group n = 88 (54.0%)	*p*
Sex, male, n (%)	116 (71.2)	50 (66.7)	65 (75.0)	0.242
Age, years, mean (SD)	65.93 ± 15.98	70.4 ± 12.35	62.2 ± 17.73	0.003
Charlson index score, median (IQR)	2 (1–3)	2 (1–4)	1.5 (0–3)	0.012
Setting: ICU versus others	69 (42.3)	34 (45.3)	35 (39.8)	0.474
Basal Glomerular Filtration Rate (eGFR), ml/min/1.73 m, median (IQR)	92.2 (75–113.5)	82.7 (49.5–98.2)	105.7 (90.2–122.9)	< 0.001
Creatinine basal, mg/dL, mean (SD)	0.89 ± 0.78	1.12 ± 0.91	0.7 ± 0.61	< 0.001
Albumin, median, mean (SD)	3.14 ± 0.75	3.14 ± 0.83	3.13 ± 0.68	0.568
Hemoglobin, mean (SD)	9.91 ± 2.01	9.45 ± 1.51	10.31 ± 2.30	< 0.001
Leucocites,cells/ml*10^3^, median (IQR)	10.3 (7.0–15.2)	9.48 (6.7–13.8)	10.9 (7.3–15.6)	0.312
Protein Reactive C, median (IQR)	119.2 (40.5–176.5)	122.1 (52.2–194.5)	102.5 (32.1–167.9)	0.134
Respiratory tract infections, n (%)	68 (41.7)	22 (29.3)	46 (52.3)	0.003
Others	95 (58.3)	53 (70.7)	42 (47.7)	
Bloodstream infection, n (%)	28 (17.2)	12 (16.0)	16 (18.2)	0.713
Target therapy, n (%)	156 (95.7)	71 (94.7)	85 (96.6)	0.546
Appropriate treatment, n (%)	25 (15.3)	7 (9.3)	18 (20.5)	0.050
**Number of associated antibiotics in combination therapy, n (%)**				
CMS in monotherapy	58 (35.6)	27 (36.0)	31 (35.2)	0.098
CMS with one antimicrobial	84 (51.5)	34 (45.3)	50 (56.8)	
CMS with two antimicrobials	21(12.9)	14(18.7)	7(8.0)	
Combination of CMS with Aminoglycosides, n (%)	17 (10.4)	6 (8.0)	11 (12.5)	0.444
Loading dose, n (%)	35 (24.5)	23 (30.7)	18 (20.5)	0.134
Maintenance dose, median (IQR)	7.5 (6–9)	9 (6–9)	6 (6–9)	0.499
Cumulative dose per patient, median (IQR)	63 (36–108)	78 (50–126)	60 (36–90)	0.013
Dose adjustment by eGFR	46 (28.2)	28 (37.3)	18 (20.5)	0.017
Duration of CMS therapy, median (IQR)	10 (6–14)	12 (7–15)	9 (6–14)	0.009
**Microorganisms, n (%)**				
* Acinetobacter* spp.	118 (72.4)	57 (76.0)	61 (69.3)	0.125
* Pseudomonas* spp.	32 (19.6)	9 (12.0)	23 (26.1)	
* Klebsiella* spp.	7 (4.3)	5 (6.7)	2 (2.3)	
* Enterobacter* spp.	3 (1.8)	2 (2.7)	1 (1.1)	
Negative results	3 (1.8)	2 (2.7)	1 (1.1)	
**Reasons for CMS discontinuation, n (%):**				
End of the treatment	94 (57.7)	36 (48.0)	58 (65.9)	–
Nephrotoxicity	14 (8.6)	14 (18.7)	0 (0.0)	
Deterioration	7 (4.3)	4 (5.3)	3 (3.4)	
Death	29 (17.8)	15 (20.0)	14 (15.9)	
Adjustment of antibiotics	17 (10.4)	6 (8.0)	11 (12.5)	
Allergic reaction	2 (1.2)	0 (0.0)	2 (2.3)	

*Nephrotoxicity was established following the Kidney Disease Improving Global Outcomes (KDIGO) classification: creatinine elevation of ≥ 0.3 mg/dL in 48 h or ≥ 1.5 times baseline creatinine in an interval of up to 7 days.

Regarding CMS prescriptions, 95.7% were prescribed as target therapy. Most of them given in combination therapy (64.4%) with one or two antimicrobials against gram-negative pathogens, 51.5% and 12.9% respectively. The antibiotics mainly used in combination therapy were carbapenems (31.0%), tigecycline (19.8%), aminoglycosides (13.5%), fosfomycin (6.3%), fluoroquinolones (7.1%) and other’s beta-lactams (22.2%). Thirty-five patients (24.5%) received a loading dose. The CMS prescriptions adjusted following variation of serum creatinine were 28.2%. Hence, CMS prescriptions were appropriate for 25 patients (15.3%). The median duration of CMS treatment was 10 days (IQR: 6–14) and the median cumulative dose per patient was 63MU (IQR: 36–108).

*A. baumannii* was the most commonly identified pathogen (72.4%) followed by *P. aeruginosa* (19.6%), *Klebsiella* spp*.* (4.3%) and *Enterobacter* spp. (1.8%), (Table 1).

75 patients (46%) developed nephrotoxicity attributable to colistin treatment, although only 14 patients (8.6%) discontinued treatment for this reason. Other reasons for withdrawing CMS treatment were cure (57.7%) followed by patient’s death (17.8%), and treatment adjustment by isolated microorganism (10.4%), among others (Table 1).

### Risk factor associated with nephrotoxicity

In the bivariate analysis of factors associated with the incidence of nephrotoxicity, the following were found: age (70.4 years vs 62.2 years, *p* = 0.003), Charlson Index (2 vs 1.5, *p* = 0.012), Basal Glomerular Filtration Rate (82.7 ml/min/1.73 m vs 105.7 ml/min/1.73 m, *p* < 0.001) and hemoglobin levels (9.5 vs 10.3, *p* < 0.001). Patients were also stratified according to baseline clearance, and those with baseline clearance below 75 ml/min/1.73 m had a higher incidence rate of nephrotoxicity (68%, *p* = 0.041) (Table 2). Furthermore, in this group of patients, the source of infection was other than respiratory (70.7% vs 47.7%, *p* = 0.003). The duration of treatment with CMS was longer (12 days vs 9 days, *p* = 0.009) and consequently the cumulative dose was higher (78 MU vs 60 MU, *p* = 0.013). Due to nephrotoxicity, a higher percentage of dose adjustment was performed in these patients (37.3% vs 20.5%, *p* = 0.017). In addition, in patients with nephrotoxicity, the rate of appropriate prescription was lower (9.3% vs 20.5%, *p* = 0.050) Table 1.

**Table 2:** Incidence of nephrotoxicity stratified by baseline creatinine clearance.

	Baseline glomerular filtration rate (eGFR), ml/min/1.73 m			
	≤ 75	75.1–92	92.1–113.5	≥ 113.6
Incidence of Nephrotoxicity, n (%)	28 (68.3)*	25 (61.0)	15 (36.6)	7 (17.5)

*p = 0.041.

Regarding clinical outcomes, the higher mortality rate corresponded to patients with nephrotoxicity (44.0% vs 27.3%, *p* < 0.001), lowest cure rate (48.0% vs 65.9% *p* < 0.001), and shorter hospital stay (45 days vs 52 days; *p* = 0.003) (Table 3).

**Table 3:** Clinical outcomes of patients treated with CMS.

Characteristics of patients	Total (n = 163)	Nephrotoxicity group n = 75 (46.0%)	Non-nephrotoxicity group n = 88 (54.0%)	HR	95%CI	*p*
Duration hospital stay, days, median (IQR)	48 (29–94)	45 (29–76)	52 (27–106)	0.992	0.986–0.997	0.003
Clinical success, n (%)	94 (57.7)	36 (48.0)	58 (65.9)	0.393	0.244–0.631	< 0.001
In-hospital mortality, n (%)	57 (35.0)	33 (44.0)	24 (27.3)	5.850	3.573–9.568	< 0.001

In the multivariate analysis, the Basal Glomerular Filtration Rate (eGFR) was a risk factor independently associated with nephrotoxicity; patients with eGFR > 90 ml/min/1.73 m had a lower risk for nephrotoxicity (HR = 0.267, 95%CI 0.161–0.443; *p* > 0.001). Higher hemoglobin levels were also a protective factor (HR = 0.898, %CI 0.793–1.015). Finally, patients with respiratory infection have a lower risk of nephrotoxicity (39% less) (HR = 0.610, 95%CI 0.362–1.025) (Table 4).

**Table 4:** Risk factors for nephrotoxicity, multivariate Cox regression analysis.

	HR	IC 95%	*p*
eGFR > 90 ml/min/1.73 m	0.267	0.161–0.443	< 0.001
Hemoglobin	0.898	0.793–1.015	0.086
Respiratory tract infections	0.610	0.362–1.025	0.062

**Figure 1:**
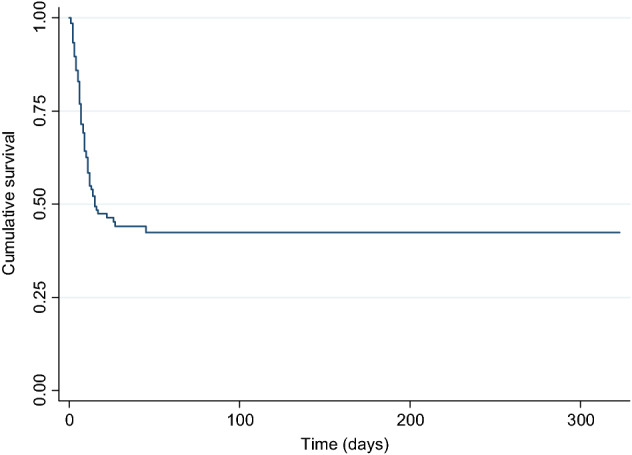
Kaplan–Meier survival curve of patients with incidence of nephrotoxicity.

Likewise, according to the Kaplan–Meier survival analysis (nephrotoxicity-free time) the probabilities of not presenting nephrotoxicity at day 7 from the beginning of the treatment were 71.5% and at day 14 were 52.5% (Fig. 1).

### Risk factor associated with in-hospital mortality

To assess the impact of nephrotoxicity on mortality, a univariate analysis was carried out. Risk factors associated with mortality were identified, including: age (HR 1.043, 95%CI 1.021–1.065, *p* < 0.001), Charlson Index (HR 1.274, 95%CI 1.116–1.454, *p* < 0.001), basal creatinine (HR 1.391, 95%CI 1.084–1.785, *p* < 0.01) and nephrotoxicity (HR 5.383, 95%CI 3.126–9.267, *p* < 0.001) (Table 5).

**Table 5:** Risk factors for all cause in-hospital mortality, univariate Cox regression analysis.

Variables	HR	IC 95%	*p*
Sex, male	0.996	0.544–1.825	0.990
Age, years, mean	1.043	1.021–1.065	< 0.001
Charlson index score	1.274	1.116–1.454	< 0.001
Setting: ICU versus others	0.731	0.428–1.246	0.249
Baseline Glomerular Filtration Rate (eGFR) (ClCrbasalCKDEPI)	0.981	0.974–0.989	< 0.001
eGFR > 90 ml/min/1.73 m	0.342	0.200–0.583	< 0.001
Creatinine basal, mg/dL	1.391	1.084–1.785	0.010
Albumin	1.164	0.832–1.629	0.375
Hemoglobin	0.919	0.789–1.069	0.274
Leucocites, cells/ml*103	1.009	0.987–1.032	0.407
Protein Reactive C	1	0.999–1.001	0.859
Respiratory tract infections	0.938	0.552–1.594	0.813
Bloodstream infection	0.951	0.480–1.884	0.886
Target therapy	2.623	0.362–19.021	0.340
Appropriate treatment	0.552	0.168–1.816	0.328
Combination therapy	0.953	0.552–1.645	0.862
2antibiotics versus gram-negative associated to CMS	1.399	0.706–2.772	0.336
Loading dose	1.212	0.576–2.550	0.613
Maintenance dose	1.013	0.890–1.153	0.846
Cumulative dose per patient	0.996	0.991–1	0.080
Mantenance dose ajustment by eGFR	1.251	0.708–2.211	0.440
Microorganisms *Acinetobacter spp.*	1.360	0.743–2.488	0.319
Nephrotoxicity	5.383	3.126–9.267	< 0.001

However, in the multivariate analysis risk factors associated with mortality were age (HR 1.031, 95%CI 1.009–1.054, *p* = 0.006) and nephrotoxicity (HR 7.266, 95%CI 2.456–7.409, *p* < 0.001) (Table 6).

**Table 6:** Risk factors for all-cause in-hospital mortality, multivariate Cox regression analysis.

	HR	IC 95%	*p*
Edad	1.031	1.009–1.054	0.006
Charlson index score	1.158	0.988–1.356	0.069
Nephrotoxicity	7.266	2.456–7.409	< 0.001

**Figure 2:**
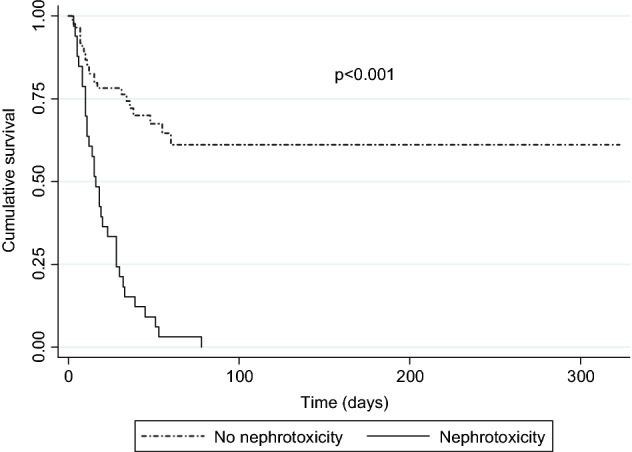
Kaplan–Meier survival curve for patients with nephrotoxicity and without (log-rank test).

According to the Kaplan–Meier survival curve, the probability of survival until day 7 was 84.8% for patients with nephrotoxicity and 91.8% for patients without nephrotoxicity. On day 14, it was 57.6% for patients with nephrotoxicity and 82.6% for patients without nephrotoxicity. Therefore, the comparative survival analysis between patients with nephrotoxicity and without it showed that patients without nephrotoxicity had a higher rate of cumulative survival (log-rank test *p* < 0.001) (Fig. 2).

## Discussion

CMS is a drug that was rarely used until recently. However, the emergence of multidrug-resistant microorganisms as carbapenemase-producing Enterobacteriaceae and the lack of therapeutic arsenal have led to its reintroduction into clinical practice.

This study shows the importance of the patient’s baseline characteristics, impact of dosage and treatment duration on the incidence of nephrotoxicity. About half of the patients developed nephrotoxicity as a result of the treatment, even though only a third of them were treated according to variations in renal clearance rates, and only a quarter received loading dosages. These results suggest that not only colistin itself but its inappropriate use is responsible for the renal function impairment rates observed.

Patients who did not develop nephrotoxicity during treatment accounted for a higher percentage of appropriate CMS prescriptions (20.5% vs 9.3%, *p* = 0.05). On the other hand, longer treatments with CMS increased the incidence of renal function alteration (9 days vs 12 days, *p* = 0.009). This may be due to CMS delay in reaching steady state for about 48 h without the loading dosage, which may, therefore, postpone the onset of clinical drug effect, increasing treatment duration and worsening the clinical outcomes of the patients^9^. Furthermore, the importance of loading dose administration in patients admitted to the ICU (in our study, these patients represent 42.3%) should be outlined due to its potential value for fast bactericidal effect^3^.

In a retrospective study including 115 patients treated with CMS, nephrotoxicity appeared in 14% of them. The dosage of CMS was adjusted to each patient’s renal clearance, and all patients undergoing continuous renal replacement therapy were excluded^18^. Nevertheless, therapeutic success was achieved in half of them, similarly to our results. Hence, specific procedures, which include variables such as eGFR and treatment duration, would make CMS most useful and less toxic. An age limit and the administration of a loading dosage also seem to be helpful to get the most out of CMS therapy.

It has been shown that nephrotoxicity is related to the accumulated dosage of CMS per patient^19^, which is also the result of our study. This could be explained by the drug’s chemical structure and its mechanism of action. Despite the high incidence of serum creatinine alteration (46%), treatment was interrupted in only 8.6% of the patients. However, in another retrospective study with a cohort of patients similar to ours, the incidence of nephrotoxicity was 60.4% (95% CI, 50.8–69.2%)^20^. Furthermore, it was previously found that low hematocrit levels have been associated with nephrotoxicity, although it is difficult to explain the relationship in critically ill patients, often with fluid overload^11^.

Multidrug-resistant *A. baumannii* was the most frequent microorganism treated with CMS. In more than half of the cases, CMS was administered in combination therapy -mostly with carbapenems and tigecycline- with poor outcomes. A study in which CMS monotherapy was compared to combination therapy with tigecycline at standard dosage^21^ found that patients receiving the association of tigecycline with a high dosage of CMS did not show the expected decrease in crude mortality rate. It should be considered that only bacteremias were analyzed, mostly with respiratory foci and with a dosage of tigecycline of 50 mg/12 h. For these indications, it is known that tigecycline does not improve clinical outcomes^22^. This may be due to two reasons: firstly, the dosage of tigecycline used in respiratory infections has shown to be insufficient and led to a higher failure rate in a clinical trial in which it was compared to Imipenem-cilastatin^23^; secondly, given its pharmacokinetics and pharmacodynamics, it is not recommended for use in bacteremia^24^. In our study, there were only 17% of bacteremias and most patients with respiratory infections treated with tigecycline received a high dosage—a 200 mg loading dose followed by 100 mg/12 h.

Another study, in which clinical outcomes were analyzed after treatment of *A. baumannii* infections, showed better results in the group treated with CMS monotherapy than in combination with meropenem^16^. In this study, the mortality rate was lower with CMS-resistant isolates due to loss of virulence compared to CMS-sensitive strains. We did not find a statistically significant relationship between combined treatment and mortality. However, patients treated with three antibiotics active against gram-negative bacteria had a higher incidence of nephrotoxicity. In addition, the presence of heteroresistance in strains sensitive to colistin should be considered, which may become resistant during treatment^25^. In order to avoid this and to ensure the effectiveness of colistin during treatment, it is crucial not to use CMS as monotherapy or at low dosages.

Considering that, no clear combination shows efficacy in this setting, especially when the second antibiotic is not active^26,27^. This fact supports the idea of the need for increased research on new antibiotics, in addition to faster approvals by the evaluating agencies of drugs that already exist and are more effective, less toxic and easier to use than CMS, such as cefiderocol^28^ or aztreonam/avibactam^29^.

Our study has some limitations. It has a retrospective design without a control group and was performed in only one medical center. Serum concentrations of CMS were not measured to verify the correlation between CMS serum levels and increased creatinine levels in blood. Moreover, daily creatinine monitoring in patients on CMS treatment is not common in our hospital. Nevertheless, we would like to underline that only a very small number of hospitals in Spain carry out the therapeutic drug monitoring of serum CMS levels to optimize PK/PD parameters and improve clinical outcomes.

On the other hand, our study includes a large number of patients enrolled during an extended time frame despite it is a single-center study. This evidence indicates that our center is experienced in the management of this drug and the impact of this drug on renal function.

In conclusion, inappropriate use and excessive duration lead to increased incidence of nephrotoxicity in patients treated with CMS. As a result of its complex management, including administration of the loading dosage and monitoring creatinine at least every 48 h, with consequent adjustments in maintenance dosage, a high percentage of patients are inappropriately treated. However, it remains to be one of the few treatment alternatives for life-threatening infections caused by multidrug-resistant gram-negative bacilli. In order to improve CMS dosages in the meantime, standardized procedures should be established to improve the use and adjustment of CMS until new molecules are discovered for treating these greater and greater prevalent infections.

## Supplementary Information

Supplementary Information.
