# Supplementary material for: Incidence of nephrotoxicity associated with intravenous colistimethate sodium administration for the treatment of multidrug-resistant gram-negative bacterial infections
Source: Sci Rep. 2022 Sep 10;12:15261. doi: 10.1038/s41598-022-19626-2 (PMC9464192; doi:10.1038/s41598-022-19626-2)
Supplement: Supplementary file 1 — Supplementary Information. [file 41598_2022_19626_MOESM1_ESM.docx]

**Supplementary data:**

**Table S1: Risk factors for Nephrotoxicity, univariate Cox regression analysis.**

| **Variables** | **HR** | **IC 95%** | **p** |
| --- | --- | --- | --- |
| Sex,male | 0.870 | 0.528-1.433 | 0.583 |
| Age,years,mean | 1.033 | 1.016-1.052 | **<0.001** |
| Charlson index score,median | 1.158 | 1.046-1.283 | **0.005** |
| Setting: ICU vs others | 0.975 | 0.606-1.569 | 0.918 |
| Baseline Glomerular Filtration Rate eGFR>90 ml/min/1.73m | 0.256 | 0.154-0.424 | **<0.001** |
| Creatinine basal,mg/dL | 1.273 | 1.071-1.514 | **0.006** |
| Albumin,median | 1.205 | 0.848-1.712 | 0.299 |
| Hemoglobin | 0.864 | 0.756-0.987 | **0.031** |
| Leucocites,cells/ml*103, median(IQR) | 1.003 | 0.978-1.028 | 0.824 |
| Protein Reactive C | 1.000 | 0.999-1.001 | 0.986 |
| Respiratory tract infections | 0.578 | 0.344-0.972 | **0.039** |
| Bloodstream infection | 1.082 | 0.568-2.060 | 0.810 |
| Target therapy | 0.657 | 0.239-1.805 | 0.416 |
| Appropriate treatment | 0.501 | 0.217-1.159 | 0.106 |
| Duration of CMS therapy | 0.986 | 0.953-1.020 | 0.420 |
| Combination therapy | 0.991 | 0.610-1.611 | 0.971 |
| 2antibiotics vs gram-negative associated to CMS | 1.719 | 0.938-3.148 | 0.079 |
| Loading dose | 1.370 | 0.827-2.271 | 0.221 |
| Maintenance dose | 1.022 | 0.914-1.141 | 0.707 |
| Cumulative dose per patient | 0.999 | 0.995-1.003 | 0.603 |
| Mantenance dose ajustment by eGFR | 1.569 | 0.966-2.548 | 0.069 |
| Microorganisms Acinetobacter | 1.172 | 0.685-2.003 | 0.562 |
